# Blood Glutathione S-Transferase-π as a Time Indicator of Stroke Onset

**DOI:** 10.1371/journal.pone.0043830

**Published:** 2012-09-17

**Authors:** Natacha Turck, Xavier Robin, Nadia Walter, Catherine Fouda, Alexandre Hainard, Roman Sztajzel, Ghislaine Wagner, Denis F. Hochstrasser, Joan Montaner, Pierre R. Burkhard, Jean-Charles Sanchez

**Affiliations:** 1 Biomedical Proteomics Research Group, Department of Human Protein Sciences, Faculty of Medicine, Geneva, Switzerland; 2 Swiss Institute of Bioinformatics, Medical University Centre, Geneva, Switzerland; 3 Department of Neurology, Geneva University Hospitals and Faculty of Medicine, University of Geneva, Geneva, Switzerland; 4 Neurovascular Research Laboratory, VHIR & Stroke Unit, Vall d'Hebron Hospital, Autonomous University of Barcelona (UAB), Catalonia, Spain; University of Cambridge, United Kingdom

## Abstract

**Background:**

Ability to accurately determine time of stroke onset remains challenging. We hypothesized that an early biomarker characterized by a rapid increase in blood after stroke onset may help defining better the time window during which an acute stroke patient may be candidate for intravenous thrombolysis or other intravascular procedures.

**Methods:**

The blood level of 29 proteins was measured by immunoassays on a prospective cohort of stroke patients (N = 103) and controls (N = 132). Mann-Whitney U tests, ROC curves and diagnostic odds ratios were applied to evaluate their clinical performances.

**Results:**

Among the 29 molecules tested, GST-π concentration was the most significantly elevated marker in the blood of stroke patients (p<0.001). More importantly, GST-π displayed the best area under the curve (AUC, 0.79) and the best diagnostic odds ratios (10.0) for discriminating early (N = 22, <3 h of stroke onset) vs. late stroke patients (N = 81, >3 h after onset). According to goal-oriented distinct cut-offs (sensitivity(Se)-oriented: 17.7 or specificity(Sp)-oriented: 65.2 ug/L), the GST-π test obtained 91%Se/50%Sp and 50%Se/91%Sp, respectively. Moreover, GST-π showed also the highest AUC (0.83) and performances for detecting patients treated with tPA (N = 12) compared to ineligible patients (N = 103).

**Conclusions:**

This study demonstrates that GST-π can accurately predict the time of stroke onset in over 50% of early stroke patients. The GST-π test could therefore complement current guidelines for tPA administration and potentially increase the number of patients accessing thrombolysis.

## Introduction

In spite of major advances obtained during the last decades to speed up diagnostic procedure, to improve patient management and to limit personal and societal impact, stroke remains one of the leading causes of death and serious long-term disability in industrialized countries. Many issues are still pending among which the determination of time after stroke onset appears especially challenging. A few studies have recently reported that diffusion-weighted imaging (DWI), T2-weithed and fluid-attenuated inversion recovery (FLAIR) MRI sequences could be useful for estimating the time of stroke onset within the first few hours [1]–[6]. However, the use of MRI as a surrogate marker for the early detection of stroke has also demonstrated a number of limitations, including low sensitivity, moderate inter-observer agreement and poor quality images [6]. In addition, immediate access to MRI machines is likely to be limited, if not impossible, in most hospitals worldwide. Consequently, to date, it is still not widely used in routine clinical practice for time determination and this is particularly problematic for thrombolysis or other, more invasive intravascular procedures, whose access is limited to the first 4.5 to 6 hours after stroke onset. In fact, depending on the countries and studies, only 1 to 10% of acute ischemic patients really benefited of recombinant tissue- plasminogen activator (rt-PA) therapy, the only established beneficial treatment for acute ischemic stroke [7]–[11]. The main reason of this low access rate to thrombolytic therapy is related to the high proportion of stroke patients (up to 25%) excluded due to an undetermined time of stroke onset [12]. The unknown time is also the main excluding factor of the treatment for all wake-up stroke patients [13]. In this context, we propose that a blood biomarker, presenting an early and significant elevation after symptom onset could complement the MRI imaging for the inclusion and management of ischemic stroke patient. It may allow rapid and accurate identification of the time of symptoms onset and consequently determine whether patients are within the appropriate therapeutic window.

Several biomarkers such as S100β [14], [15], glial fibrillary acidic protein (GFAP) [16], [17], matrix metalloproteinase-9 (MMP-9) [18], [19], and neuron-specific enolase (NSE) [20], [21] have been extensively studied in stroke. Our group recently explored the *post-mortem* cerebrospinal fluid (*pm* CSF) protein content, used as a model of massive brain injury, and identified new brain damage biomarkers [22]–[24], including the heart-fatty acidic binding protein (HFABP) [25], ubiquitin fusion degradation protein 1 (UFD-1) [26], PARK7 (also called DJ-1) and nucleoside diphosphate kinase A (NDKA). These new biomarkers were tested and proved promising as stroke biomarkers [27], yet none has been used in the routine assessment of stroke patients thus far.

The main objectives of the present study were to report the clinical performances of 29 molecules (glutathione S transferase π (GST-π), PARK7, H-FABP, NDKA, UFD1, S100β, Troponin I, p-Selectin, E-selectin, MMP-1, MMP-3, C-reactive protein (CRP), N-terminal pro brain natriuretic peptide (NT-proBNP), vascular cell adhesion protein (VCAM), intracellular cell adhesion protein (ICAM), interleukin (IL)-1 recepteur antagonist (ra), IL-1b, IL-6, IL-8, IL-9, IL-10, C-X-C motif chemokine 10 (CXCL-10, also called IP-10), granulocyte colony-stimulating factor (G-CSF), interferon-γ (IFN-γ), monocyte chemotactic protein-1 (MCP-1), macrophage inflammatory protein 1-α (MIP-1α), MIP-1β, vascular epidermal growth factor (VEGF), tumor necrosis factor-α (TNF-α)), to assess their performance at detecting very early stroke patients within the therapeutic window for thrombolysis.

## Patients and Methods

### Cohort 1

Over a period of two years (from December 2005 to January 2008), a total of 1721 individuals reached the emergency unit of the Geneva University Hospital with a suspected diagnosis of stroke and were managed according to our routine stroke program. Recruitment was designed to be as unbiased as possible (no age-, gender-, race- or origin-based decision to decline patient's enrolment). To be eligible for enrolment in the study, stroke patients had to meet specific inclusion and exclusion criteria.

Inclusion criteria were: 1/to present symptoms and signs suggestive of an acute or sub-acute stroke between 0 and 72 hours after onset; 2/to be hospitalized for this problem or, if stroke occurred during an ongoing hospitalization, for another unrelated problem; 3/to accept and sign the information and consent forms of the study (patient or family relatives).

Exclusion criteria were: 1/to have presented a stroke event between 72 hours and 3 months (207 subjects, 12.1%); 2/to not comply with the need of a hospitalization and/or the requirements of the study (327 subjects, 18.8%); 3/to die early after admission (5 subjects, 0.3%); 4/to refuse to sign information and consent forms (86 subjects, 5.0%); 5/to be unable to determine accurate time of stroke onset (555 subjects, 32.2%); 6/to present with the following stroke-related conditions: sub-dural or epidural hematoma, subarachnoidal haemorrhage, traumatic brain concussion, transient global amnesia (79 subjects, 4.6%); 7/to present with the following concomitant medical conditions: metastatic cancer, liver cirrhosis, chronic or acute renal failure rand recent myocardial infarction (242 subjects, 14.1%); 8/to present a significant psychiatric condition, which may interfere with study requirements, including severe depression, severe or uncontrolled bipolar disorder, schizophrenia or other severe psychoses (27 subjects, 1.6%); 9/to be treated with medications known to significantly interfere with brain function, including antipsychotics and lithium (2 subjects, 0.1%).

Altogether, a total of 209 stroke patients (representing 12.1% of all assessed patients) were finally included, from which we specifically selected for this pilot study all patients (N = 103) having had a unique blood draw within the first 36 h after symptom onset (ischemia: 66, hemorrhage: 9, transient ischemic attack (TIA): 19 and stroke with unknown origin (SUO): 9).

All these stroke patients followed a standardized protocol of clinical and neuro-radiological assessment and therapeutic interventions, which were supervised by trained neurologists from the Neurology Department. Most patients were managed in the stroke unit of our department where they had a daily neurological examination (or more frequently when needed) and repeated measures of blood pressure, pulse, temperature, partial pressure of oxygen, ECG, blood glucose, dyslipidemia (defined as increased lipids concentrations: cholesterol >200 mg/dL or triglyceridemia >200 mg/dL, self-reported history and/or any treatment for dyslipidemia). A detailed assessment of their vascular risk factors was also available. In addition, they underwent a workup including one to several brain CT-scan(s), with angiographic sequences at admission and one to two brain MRI scan(s).

During the same period, control subjects were also enrolled in the study. Two subgroups were defined: a non-neurological disease group (NND, N = 62) which included patients suffering from various types of medical or surgical conditions and healthy patients (typically patient's family relatives) and a group with other neurological disease (OND, N = 57) which included subjects suffering from various neurological conditions, outside stroke and cerebrovascular diseases, such as migraine, epilepsy, neuropathy, myopathy and neurodegenerative diseases (Parkinson's disease, Alzheimer's disease, ALS). A mimicking stroke condition group (Mim, N = 13) was also set with patients who were diagnosed as suspected stroke at admission and finally reclassified as vertigo (N = 4), seizure (N = 4), headache (N = 4) and contusion (N = 1). In these control groups, patients were required not to have a past or present history of stroke, cerebrovascular disease, coronaropathy or any atherosclerotic or thrombotic diseases. Among the recruited control patients, 132 patients, who did not significantly differ from stroke patients with respect to age and gender, were included in this study.

### Cohort 2

One hundred consecutive patients with an acute ischemic stroke admitted to the emergency department of the Vall d'Hebron Hospital (Barcelona, Spain) within the first 3 h after symptoms onset were recruited from 2006 to 2007. Stroke onset was defined as the last time the patient was known to be asymptomatic. All patients received intravenous t-PA in a standard 0.9 mg/Kg dose (10% bolus, 90% continuous infusion during 1 h). Each patient included in the study underwent a standardized protocol of clinical and neuroradiological assessments as previously described [28].

Demographic and risk factors data were summarized in Tables 1 and 3. The local ethical committees approved the study (Cohort 1: N.A.C. (Neuclid, Apsic, Chirurgie) Ethical committee of the Geneva University Hospitals, CER05-026 (05-058); Cohort 2: PR(HG)89/2003). In accordance with the Helsinki Declaration, written informed consent was obtained from control patients, diseased patients or patient's relatives.

**Table 1:** General characteristics of the 2 cohorts.

	Cohort 1		Cohort 2
	Controls	Stroke	Stroke
n	132	103	155
Age			
Mean ± SD	64.2±15.0	66.7±14.5	72.2±11.0
Median (Min-Max)	67.5 (25–88)	67.0 (33–89)	74.00 (45–97)
Gender			
Male n (%)	76 (57.6)	57 (55.3)	86 (55.5)
Time onset of symptoms Median			▵
(Min.)Mean ± SD		896.4±617.0	
(Range)		960.0 (5.0–2130.0)	
NIHSS basal			
0–7, n (%)		62 (70.5)	5 (3.2)
>7, n (%)		26 (29.5)	150 (96.8)
NA		15	
Atrial Fibrillation			
Yes, n (%)		33 (32.0)	62 (40.0)
NA			4
Hypertension			
Yes, n (%)		65 (63.1)	NA
Alcohol			
Yes, n (%)		2 (1.9)	NA
Tobacco			
No, n (%)		58 (56.3)	115 (74.2)
NA			13
Diabetes Mellitus			
Yes, n (%)		13 (12.7)	50 (32.2)
NA			6

NA: not available.

▵ : All patients were collected within the first 3 h after symptom onset.

### Blood collection and sampling

Blood samples were collected in dry tubes according to standard operating procedures (SOP) [29]. After centrifugation (1000 g for 10 min) to discard blood cells, serum samples were immediately aliquoted and stored at −80°C until analysis. All the samples presenting a hemolytic aspect were excluded of immunoassay analyses.

### Sandwich ELISA immunoassay procedures

Home-made sandwich enzyme linked immunosorbent assay (ELISAs) were performed to quantify NDKA, DJ-1, UFD-1 and GST-π in sera as previously described [27], [30], [31]. The 25 other proteins were quantified with commercial ELISA kits (for details, see Supporting Information S1 and S2) according to the manufacturer's recommendations. All tests were done blindly by two experimented lab technicians.

For all the immunoassays, each sample was assessed in duplicates and distributed randomly on the 96 micro-titer plates. Each molecule selected in this study was tested for the first time on these patient samples and the results have never been reported before. The reproducibility of the tests was assessed with the coefficient of variation calculated on the duplicates samples (inter-run) and on the calibration curves and quality control samples (intra-run). The intra and inter-run coefficients of variation were below 10% for all sets.

### Statistical analysis

Between group differences were tested using the non-parametric Mann-Whitney U test (2 groups). To account for multiple testing, Bonferroni correction was used as post hoc test when a significant result was obtained in order to adapt the p-values by the number of biomarkers tested for each analysis.

The diagnostic performances of the biomarkers were assessed by building receiver operating characteristics (ROC) curves. For each molecule, thresholds were chosen either as the maximal sum of sensitivity (Se) and specificity (Sp) (Youden's index), or as the best cut-offs depending on preference for high Se (>90%) or high Sp (>90%). Associated negative and positive predictive values were also calculated. Diagnostic odds ratios (ORs) were calculated as follows (positive likelihood ratio (LR) (Se/(1-Sp))/negative LR ((1-Se)/Sp) according to [32]. Power tests were performed using the pROC tool [33].

Statistical analyses were performed using IBM SPSS Statistics version 19.2 (SPSS Inc., Chicago, IL, USA) and TIBCO Spotfire S+® version 8.2 (TIBCO software Inc., Palo Alto, CA, USA) softwares.

## Results

### Serum concentration of the 29 molecules in early and late stroke patients

Twenty nine molecules (GST-π, PARK7, H-FABP, NDKA, UFD1, S100β, Troponin I, p-Selectin, E-selectin, MMP-1, MMP-3, CRP, NT-proBNP, VCAM, ICAM, IL-1 ra, IL-1b, IL-6, IL-8, IL-9, IL-10, CXCL-10, G-CSF, IFN-γ, MCP-1, MIP-1α, MIP-1 β, VEGF and TNF-α) were tested by immunoassays on the blood samples of cohort 1 (132 control and 103 stroke patients with time after symptom onset ≤36 h, general description in Table 1) and classified according to the significance obtained for concentration differences between control and stroke patients (Mann-Whitney U tests, Table 2). Blood elevation of GST-π, DJ-1, NT-proBNP, NDKA and IL6 in stroke patients were highly significant (*p<.0001*). H-FABP, IL-1ra, IL-8, S100β, CRP and Troponin I concentrations were also significantly increased whereas MMP3 and E-selectin concentrations showed a reduction in stroke patients compared to controls (*0.001<p<0.05*). Finally, all the 16 remaining molecules showed no difference in blood concentration between stroke and control patients. Further analyses were restricted to the 5 best molecules (GST-π, DJ-1, NT-pro-BNP, NDKA and IL-6).

**Table 2:** blood concentration of the 29 biomarkers between stroke (N = 103) and control patients (N = 132) in cohort 1.

Molecules	Controls	Stroke	p value*
	Mean± SD	Mean± SD	
	Median (Min.- Max.)	Median (Min.- Max.)	
**GST-π**	**17.3±9.8**	**41.6±51.3**	**<.001**
	**15.2 (6.1–60.6)**	**20.1 (4.8–320.1)**	
**DJ1**	**74.9±140.1**	**161.7±355.7**	**<.001**
	**39.7 (0–1399.0)**	**69.9 (2.8–3036.8)**	
**NDKA**	**5.5±5.8**	**15.8±36.7**	**<.001**
	**3.9 (0.5–47.0)**	**5.8 (0.0–276.6)**	
**NT-proBNP**	**316.0±639.9**	**1244.9±2180.2**	**<.001**
	**115.5 (20.0–5180.0)**	**416.0 (20.0–10776.0)**	
**IL-6**	**31.1±209.9**	**97.0±497.0**	**<.001**
	**4.3 (0.0–2108.6)**	**7.0 (0.0–4366.9)**	
MMP-3	17.7±15.5	14.0±14.6	.008
	13.4 (1.7–104.0)	10.7 (0.0–12.2)	
UFD-1	7.3±20.4	7.7±17.1	0.01
	2.1 (0.0–147.0)	2.8(0.0–143.0)	
IL-1ra	76.1±234.2	312.5±1321.4	.012
	15.2 (0.0–1758.4)	34.0 (0.0–9411.7)	
IL-8	6.4±69.0	65.4±366.7	.015
	0.0 (0.0–786.3)	0.0 (0.0–3349.9)	
S100β	12.5±34.4	40.3±87.9	.015
	0.0 (0.0–339.4)	4.65 (0.0–505.6)	
CRP	7.2±35.7	8.1±14.7	.017
	1.8 (0.0–400.0)	3.12 (0.0–107.7)	
Troponin I	40.3±24.7	102.6±674.4	.017
	20.0 (0.0–2790)	20.0 (0.0–685.0)	
H-FABP	2.1±2.1	4.1±8.0	.024
	1.5 (0.2–15.7)	1.8 (0.07–74.1)	
E-selectin	42.5±30.9	34.5±16.2	.046
	35.7 (11.7–233.5)	32.5 (10.9–98.2)	
VEGF	127.8±812.1	131.3±282.0	0.15
	15.7 (0.0–9261.5)	32.1 (0.0–2177.8)	
G-CSF	28.2±128.6	35.6±106.6	0.17
	12.6 (0.0–1470.2)	15.9 (0.0–996.8)	
IL-9	50.6±326.3	74.8±262.3	0.18
	1.0 (0.0–3670.8)	1.7 (0.0–1575.1)	
IL-10	72.1±311.7	77.8±199.3	0.22
	38.6 (0.0–3571.9)	42.9 (0.0–1833.1)	
IFN-γ	17.9±79.0	59.3±269.6	0.22
	4.0 (0.0–715.0)	5.6 (0.0–2464.8)	
VCAM	620.2±200.4	663.9±241.1	0.29
	589.8 (95.5–1321.5)	615.0 (283.7–1409.0)	
IL-1b	2.6±13.5	24.9±206.7	0.35
	0.8 (0.0–125.1)	0.8 (0.0–2087.6)	
P-Selectin	119.0±52.6	151.0±131.9	0.37
	117.7 (37.4–364.0)	115.7 (35.4–812.1)	
MMP-1	5.0±5.7	4.2±4.0	0.37
	3.5 (0.3–47.0)	3.5 (0.1–26.6)	
IP-10	784.1±908.3	732.6±779.5	0.65
	510.4 (15.5–8016.1)	500.3 (0.0–4747.1)	
MIP-1b	132.1±112.1	143.3±206.8	0.67
	109.8 (5.0–1032.9)	110.7 (3.7–2005.4)	
ICAM	270.1±139.9	254.1±101.1	0.68
	257.7 (47.2–1117.5)	250.4 (83.4–566.7)	
TNF-α	24.2±147.9	72.0±349.7	0.72
	6.1 (0.0–1598.0)	6.4 (0.0–2925.5)	
MIP-1a	65.7±747.3	10.6±86.5	0.76
	0.0 (0.0–8521.2)	0.0 (0.0–873.4)	
MCP-1α	84.8±80.4	104.2±241.0	0.91
	63.6 (5.3–514.6)	62.6 (7.8–2397.7)	

* Significant concentration was set at 0.002 after Bonferroni correction (Mann-Whitney U tests, two-tailed tests).

The stroke population (cohort 1) was dichotomized according to the time of symptom onset, less or equal to 3 h after stroke for the early group (N = 22) and strictly above 3 h for the late group (N = 81, Table 3). Except for the NIHSS (National Institutes of Health Stroke Scale) score at admission, no age, sex and risk factor differences were observed between early and late stroke patients.

**Table 3:** Detailed characteristics of the stroke patients (cohort 1).

	Stroke patients		
	Early	Late	p-value†
n	22	81	
Subtypes			
Hemorrhagic	2	7	
Ischemic	16*	50**	
TIA	1	18	
SUO	3	6	
Age			0.28
Mean ± SD	69.9±14.0	65.8±14.6	
Median (Min-Max)	72.0 (36.0–89.0)	66.0 (33.0–89.0)	
Gender			0.47
Male, n (%)	14 (63.6)	43 (53.1)	
Time onset of symptoms (Min.)			
Mean ± SD	120.0±42.8	1107.2±523.6	
Median (Range)	130.0 (5.0–180.0)	1440.0 (190.0–2130.0)	
NIHSS basal			<.0001
0–7, n (%)	7 (33.3)	55 (82.1)	
>7, n (%)	14 (66.7)	12 (17.9)	
Atrial Fibrillation			0.32
Yes, n (%)	9 (40.9)	24 (29.6)	
Hypertension			0.45
Yes, n (%)	12 (54.5)	53 (65.4)	
Tobacco			0.45
No, n (%)	10 (45.5)	48 (59.3)	
Diabetes Mellitus			0.48
Yes, n (%)	4 (18.2)	9 (11.1)	

† : Fischer exact tests.

* Among them, 3 patients presented initially an ischemia and a hemorrhage as a secondary event.

** Among them, 7 patients presented initially an ischemia and a hemorrhage as a secondary event.

The 5 molecules were classified according to the significance obtained for concentration differences between early and late stroke patients (Mann-Whitney U tests, Table 4). Blood GST-π concentrations showed a highly significant decrease between early (mean± SD: 75.6±57.1) and late stroke patients (mean (ug/L) ± SD: 32.3±45.7, *p≤.0001*) and no influence of severity was observed in the elevation of GST-π in the early stroke patients (NIHSS 0–7, N = 62 stratified Wilcoxon test, p = 0.011; NIHSS>7, N = 26, stratified Wilcoxon test, p = 0.003). NDKA and PARK7 concentrations were slightly reduced in late stroke patients (NDKA in early patients mean (ug/L) ± SD: 24.0±57.3; late patients mean± SD: 13.5±28.8; PARK7 early patients mean (ug/L) ± SD: 215.3±313.5; late patients mean± SD: 147.0±366.9). Finally, no significant difference was observed for blood NT-proBNP and IL6 concentrations between early vs. late stroke patients. For all these molecules, when considering only the ischemic sub-type, similar significant differences between early and late patients were obtained (data not shown).

**Table 4:** Blood concentration of the 5 best molecules in early and late stroke patients.

Molecules	Early stroke	Late stroke	p value*
	Mean± SD	Mean± SD	
	Median (Min.- Max.)	Median (Min.- Max.)	
**GST-π**	**75.6±57.1**	**32.3±45.7**	**≤.001**
	**64.7 (14.2–187.8)**	**17.9 (4.8–320.1)**	
**NDKA**	**24.0±57.3**	**13.5±28.8**	**0.009**
	**8.4 (2.9–276.6)**	**5.2 (0.0–208.6)**	
**DJ-1**	**215.3±313.5**	**147.0±366.9**	**0.021**
	**95.5 (18.6–1439.1)**	**61.4 (2.8–3036.8)**	
NT-proBNP	725.4±1020.1	1385.9±2385.9	0.36
	299.0 (20.0–4251.0)	429.0 (20.0–10776.0)	
IL-6	21.0±28.5	117.6±559.2	0.18
	9.8 (0.0–110.1)	6.8 (0.0–4366.9)	

* Significant concentration was set at 0.01 after Bonferroni correction (Mann-Whitney U tests, two-tailed tests).

**Figure 1:**
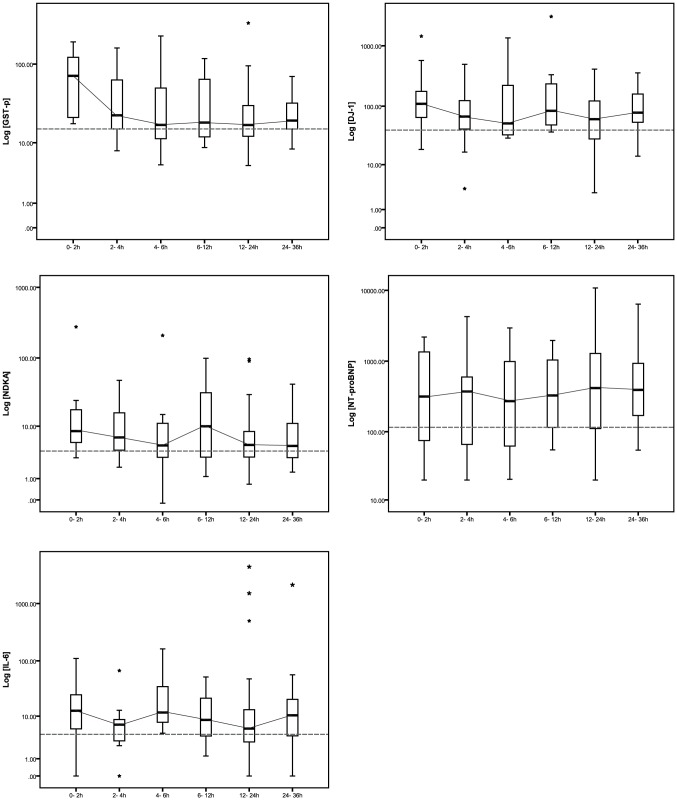
Time course of the blood concentrations of GST-π, NDKA, DJ-1, NT-proBNP and IL-6 after stroke onset. The grey dashed lines correspond to the median concentration of the molecules in the control patients. The grey lines connect the median of the box plots. 0–3 h: N = 22, 3–6 h: N = 15, 6–12 h: N = 8, 12–24 h: N = 45 and 24–36 h: N = 13. Note that there is no overlap between the different subgroup of times and that each patient has only one blood sampling within the 36 h after stroke onset.

The influence of the delay between onset of symptoms and blood sampling on these molecule concentrations was also investigated by stratifying samples (one per patient) into 5 groups (Figure 1). Significant elevation of GST-π, NDKA and DJ-1 concentrations were already observed in the blood of early stroke patients (blood collected within the first 3 h after stroke onset, N = 22) compared to patients sampled between 3 and 6 h. Indeed, patients sampled after 3 h displayed concentration of GST-π, NDKA, DJ-1 close to normal levels whereas the IL-6 concentration displayed a slight but significant second increase at 24 h after stroke onset. No early change in NT-proBNP concentrations was detected in the blood of stroke patients compared to the controls. The NT-proBNP elevation occurred only later, above 12 h after stroke onset and remained elevated up to 36 h post-stroke. The performances of GST-π, NDKA and DJ-1 for discriminating early and late stroke patients were reported in Table 5. GST-π was the only molecule to have a significant area under the curve (AUC: 0.79).

**Table 5:** Clinical performances of the 3 best molecules for discriminating early (blood sampling between 0 and 3 h after symptoms onset, N = 22) vs. late stroke patients (blood sampling strictly after 3 h, N = 81).

		GST-π	NDKA	DJ-1
	AUC (95%CI)	0.79*(0.69–0.89)	0.68 (0.57–0.79)	0.66 (0.54–0.78)
**Youden index**	CO (ug/L)	39.6	4.4	60.3
	Se/Sp (%)	68.2/82.5	91.0/44.0	86.4/50.0
	OR	10.1	8.0	6.4
	NPV/PPV (%)	90.4/51.7	94.7/30.8	93.0/32.2
**High Se**	CO (ug/L)	17.7	4.4	50.8
	Se/Sp (%)	90.9/50.0	91.0/44.0	90.9/42.5
	OR	10.0	8.0	7.4
	NPV/PPV (%)	95.2/33.3	94.7/30.8	94.4/30.3
**High Sp**	CO (ug/L)	65.2	23.1	443.0
	Se/Sp (%)	50.0/91.2	18.2/90.1	18.2/97.5
	OR	10.4	2.0	8.6
	NPV/PPV (%)	86.9/61.1	80.2/33.3	81.2/66.7

NPV: negative predictive value/PPV: positive predictive value.

* The AUC of GST-π was significantly better than AUC of NDKA and DJ-1 (p = 0.034 and 0.020 respectively). No significant difference was obtained between AUC of NDKA and of DJ-1 (p = 0.63).

An AUC above 0.74 was estimated as significant (significance 0.05 and power 0.95, made with Power tests/Sample size item from pROC software).

### Early molecule concentration elevation as indicator of tPA eligibilty

Due to their early elevations in the blood of stroke patients and because time is one of the crucial parameters for tPA administration, the performances of GST-π, NDKA and DJ-1 were also investigated for discriminating patients treated with tPA (early ischemic and SUO patients, N = 12) *vs.* ineligible patients (late ischemic, TIA and stroke-mimicking condition patients, N = 102). In this context, the GST-π test similarly emerged as the best biomarker (AUC: 0.83). Results were reported in Table 6.

**Table 6:** Clinical performances of the molecules for discriminating tPA treated (N = 12) vs. ineligible patients (N = 104).

		High Se			High Sp		
Molecules	AUC	CO	Se/Sp	OR	CO	Se/Sp	OR
	(95%CI)	(units)	(%)		(units)	(%)	
GST-π	0.83*	17.7	91.7/45.6	9.3	65.2	58.3/89.3	11.7
	(0.71–0.95)						
NDKA	0.69	4.4	91.7/40.8	8.6	23.1	16.7/89.3	1.7
	(0.57–0.82)						
DJ-1	0.62	50.8	91.7/37.3	6.6	443.0	8.3/95.1	1.7
	(0.48–0.75)						

* The AUC of GST-π was significantly better than AUC of DJ-1 (p = 0.016). No significant difference was obtained between GST-π vs. NDKA (p = 0.11) and NDKA vs. DJ-1 (p = 0.14).

An AUC above 0.80 was estimated as significant (significance 0.05 and power 0.95, made with Power tests/Sample size item from pROC software).

As an independent verification set, the GST-π test was verified retrospectively on cohort 2 comprising only early ischemic patients (≤3 h) treated with tPA (general description of this cohort shown in the Table 3). Interestingly, the promising results obtained on cohort 1 were confirmed in this independent cohort. Indeed, 98 out of 100 ischemic stroke patients treated with tPA presented a GST-π concentration above the Se oriented threshold previously defined (17.7 ug/L) and were consequently detected as positive (mean± SD: 163.0±435.3, median (min-max): 81.5 (16.9–3913.5). On the other side, if the Sp oriented cut-off value was preferred (65.2 ug/L), 70% of the Spanish stroke patients displayed blood GST-π level above this value at admission.

## Discussion

The main objective of this study was to evaluate clinical performances of GST-π for predicting time of stroke onset and its potential utility for maximizing patient access to thrombolytic therapy. Interestingly, compared to 28 molecules classically described in stroke research, GST-π emerged as the best biomarker with promising clinical performances associated with an early elevation within the first 3 h after stroke onset and a potential interest for discriminating eligible vs. ineligible patients for thrombolytic therapy.

The results presented here clearly showed that GST-π concentration increased in the blood of stroke patients almost immediately after the stroke event. Elevation occurred within the first 3 h after symptom onset – even within less than 1 h in some patients. More importantly it decreases quickly after 3 h (late stroke patients) to finally reach a normal level 6 h after symptom onset. A similar although less consistent pattern was observed for NDKA and PARK7, confirming previous results [27].

Many studies investigated the potential interest of new blood tests based on blood biomarkers to speed up and improve the detection of stroke patients. However none of the candidate biomarkers has reached routine use at bedside so far. In this context and considering that no accurate clinical test currently exist to predict the time of stroke onset and that an undetermined time of stroke (more than 30% in our study) represents one the most common excluding factor for accessing thrombolysis protocols, the GST-π test appears as a novel and promising tool for this purpose. These data support GST-π as a very early marker of stroke, a feature that makes it highly valuable to include a stroke patient within the therapeutic window currently defined for IV or IA thrombolysis and other invasive endovascular interventions. Indeed, as shown in our cohorts where international clinical criteria for tPA administration were used, GST-π is able to discriminate treated vs. ineligible patients. Indeed, using the same cut-offs, we obtained similar performances in two independent cohorts: 98% of Spanish ischemic patients presented GST-π level superior to 17.7 ug/L compared to 90% in the Swiss cohort and the GST-π level was above 65.2 ug/L for 70% of the Spanish patients (compared to 50% for the Swiss cohort). In our study, the determination of the time window was restricted to the first 3 h after stroke onset and not to the 4.5 h often proposed for tPA administration for two main reasons. The first cause was that our cohort was collected before the European Agency accepted an extension of the therapeutics window to 4.5 h and, to date, this extended time window is not yet accepted in all countries. The second reason was that we considered that the time needed for testing GST-π at the central core laboratory (30 to 45 min) should be included in the tPA therapeutic window.

The ability of GST-π to predict the time window after stroke onset and consequently the potential eligibility for thrombolytic therapies may open new avenues for the management of stroke patients but also more specifically for the wake-up stroke patients, representing up to 25% of stroke events and traditionally excluded from treatment because of unknown time of symptom onset

GST-π belongs to a family of multifunctional enzymes involved in the detoxification processes and therefore provides protection to cells exposed to oxidative stress or chemical reagents. GST-π presents a wide tissue expression pattern and has been reported to be present in the central nervous system [34], with a strong expression in different areas of the brain [35]–[37] and also in brain capillaries [38]. Although GST-π expression is relatively ubiquitous, its massive blood elevation following stroke was, to our knowledge, not reported to date. Thus, these results established GST-π as a reliable indicator of anoxic and/or cerebrovascular injury.

Several hypotheses regarding the mechanisms by which GST-π may gain access to and be over-expressed in the blood of brain injury related patients can be proposed. Soluble proteins produced by an injured neuron or glial cell may be released in the extra-cellular space surrounding brain cells, access CSF through the relatively permeable ependymal and pial barriers and eventually appear in the blood via the arachnoid villi. Similarly, the proteins may enter the CSF through the CSF containing Virchow-Robin perivascular spaces. However, these indirect routes require several hours before the proteins reach the blood and therefore do not provide a satisfactory explanation for the rapid increase of GST-π in the blood of stroke patients (<3 hours). Another possibility involves the direct release of these proteins through altered micro-vessels, notably capillaries and venules, within or in the vicinity of the lesion and their appearance in systemic blood flow. Furthermore, the rapid decrease of GST-π expression after 3 h could be related to the short half-life of this protein [39].

### Study limitations

As for all biomarkers, a number of issues remain to be solved before the GST-π test can be validated, recognized by the scientific community and used in clinical practice. The main limitation of this study relates to the limited number of early stroke patients studied in the original cohort, yet this problem was somehow circumvented by the confirmation of the GST-π performances on a larger (N = 100) and independent cohort. Evaluating GST-π in extended, prospective and multi-center cohorts is now required. In addition, the kinetic of GST-π as function of time after stroke onset presented in our study was established without sequential measurements of this biomarker in individual patients. Even if preliminary data obtained on 3 patients in our cohort having 3 sequential blood draws during the first 24 h after admission were consistent with a high level of GST-π followed by a rapid decrease after 3 to 6 hours (data not shown), this result needs confirmation on a larger longitudinal cohort to definitively assess the pattern of GST-π in ischemic stroke patients.

Secondly, the stroke patients included in our study exhibited typical and well-defined manifestations of stroke. It remains to be established how this biomarker will behave in case of atypical presentations with vague symptoms. Finally, considering the stroke severity as potential cofounding factor for interpreting GST-π results (NIHSS at admission was significantly different between early and late stroke patients), we found no influence of severity on the GST-π time course. Nevertheless, due to the small sample size, classical adjustment with logistic regression was not performed and therefore, it appears premature to definitively close this question.

In conclusion, we believe that this study strongly supports a potential added value of GST-π for the management of stroke patients. The test is likely to increase significantly the number of patient having access to thrombolysis and which are excluded at the moment because of uncertainty surrounding time of stroke onset.

## Supporting Information

Supporting Information S1 **Home-made sandwich ELISA immunoassay procedures.**
(DOCX)

Supporting Information S2 **Summary of the 29 immunoassay tests used in this study.**
(DOCX)
