# Supplementary material for: Blood Glutathione S-Transferase-π as a Time Indicator of Stroke Onset
Source: PLoS One. 2012 Sep 17;7(9):e43830. doi: 10.1371/journal.pone.0043830 (PMC3444482; doi:10.1371/journal.pone.0043830)
Supplement: Supporting Information S1 — Home-made sandwich ELISA immunoassay procedures. (DOCX) [file pone.0043830.s001.docx]

**Option : Supplemental data 1**

# Home-made sandwich ELISA immunoassay procedures

50 μL of appropriate diluted antibody-biotin conjugated (2 μg/mL) (Biosite, California, USA) were incubated one hour at 37°C in 96-well Reacti-Bind^TM^ NeutrAvidin^TM^ coated Black Plates (Pierce, Rockford, IL). After the washing steps, 50 μL of antigen (NDKA: Biosite, California, USA or GST-π: Sigma, Missouri, USA) or serum with appropriate dilution (1/2 and 1/10 for NDKA and GST-π respectively) was added and incubated for one hour at 37°C. Excess of recombinant was removed by washing and 50μL of alkaline phosphatase conjugated antibodies (Biosite, California, USA) at 2mA/mL were added for one hour at 37°C. The 96-well plate was then washed and 50 μL of fluorescence Attophos^®^ AP Fluorescent substrate (Promega, Madison, WI) were added. Plates were read immediately on a SpectraMax GEMINI-XS, fluorometer microtiter plate reader (Molecular Devices Corporation, Sunnyvale, CA, U.S.A.) using the kinetic mode relative fluorescence units (RFU) (λ_excitation_=444 nm and λ_emission_=555 nm). The NDKA and GST-π recombinant proteins were diluted to concentrations of 100, 50, 25, 12.5, 6.25, 3.125, 1.56 and 0 µg/L in the dilution buffer. A calibration curve was performed using a linear regression in the linear range of the curve (1.56 to 100 µg/L). Protein levels were initially expressed in RFU and the concentrations were calculated *via* the calibration curve. The precision of these immunoassays was in accordance with the general accepted recovery values ranging from 75 and 125%.
