# Supplementary material for: Blood Glutathione S-Transferase-π as a Time Indicator of Stroke Onset
Source: PLoS One. 2012 Sep 17;7(9):e43830. doi: 10.1371/journal.pone.0043830 (PMC3444482; doi:10.1371/journal.pone.0043830)
Supplement: Supporting Information S2 — Summary of the 29 immunoassay tests used in this study. (DOCX) [file pone.0043830.s002.docx]

**Supplemental data 2**

*Summary of the 29 immunoassay tests used in this study.*

| **ELISA type**  **Compagnies** | **Molecules** | **References** | **Units** |
| --- | --- | --- | --- |
| Beads array technology |  |  |  |
| R&D Sytems | Human Adhesion Molecule Base Kit Bioplex | LAD000 |  |
|  | sICAM-1/CD54 | LAD720 | pg/ml |
|  | sVCAM-1/CD106 | LAD809 | pg/ml |
|  | sE-Selectin/CD62E | LAD724 | pg/ml |
|  | sP-Selectin/CD62P | LAD137 | pg/ml |
|  |  |  |  |
|  | Human MMP Base kit Bioplex | LMP000 |  |
|  | MMP-1 | LMP901 | pg/ml |
|  | MMP-3 | LMP513 | pg/ml |
|  |  |  |  |
|  | Human Obesity Base Kit Bioplex | LOB000 |  |
|  | C-Reactive Protein (CRP) | LOB1707 | mg/l |
|  |  |  |  |
| Bio-Rad | Human Cytokine Base Kit Bioplex |  |  |
|  | Hu IL-1b | 171-B12832 | pg/ml |
|  | Hu IL-1ra | X5000B6AZ1 | pg/ml |
|  | Hu IL-6 | 171-B10719 | pg/ml |
|  | Hu IL-8 | 171-B11054 | pg/ml |
|  | Hu IL-9 | X5000MCLD2 | pg/ml |
|  | Hu IL-10 | 171-B11356 | pg/ml |
|  | Hu G-CSF | 171-B14357 | pg/ml |
|  | Hu IFN-γ | 171-B11921 | pg/ml |
|  | Hu IP-10 | X5002JFFH8 | pg/ml |
|  | Hu MCP-1(MCAF) | 171-B14653 | pg/ml |
|  | Hu MIP-1α | X50053UUYG | pg/ml |
|  | Hu MIP-1β | 171-B14918 | pg/ml |
|  | Hu TNF-α | 171-B12236 | pg/ml |
|  | Hu VEGF | X50005KMT | pg/ml |
| Home-made ELISA sandwich |  |  |  |
| Biosite | DJ-1 |  | ng/ml |
|  | GSTP |  | ng/ml |
|  | NDKA |  | ng/ml |
|  | UFD1 |  | ng/ml |
|  |  |  |  |
| ELISA Sandwich |  |  |  |
| Hycult®Biotech. | H-FABP | HK401-402 | pg/ml |
|  |  |  |  |
| Abnova Corp. | S100B | KA0037 | pg/ml |
|  |  |  |  |
| Siemens Comp. | NT-proBNP | Immulite 2000 | pg/ml |
|  |  |  |  |
| Dade | Troponin-I | Stratus Analyzer | ug/L |
